# Sustainable Chemical Approaches for Wastewater Treatment and Resource Utilization

**DOI:** 10.3390/molecules31203644

**Published:** 2026-10-13

**Authors:** Jingtao Bi

**Affiliations:** Engineering Research Center of Seawater Utilization of Ministry of Education, School of Chemical Engineering and Technology, Hebei University of Technology, Tianjin 300401, China; jingtaob@gmail.com or jingtaob@hebut.edu.cn

Water and wastewater treatment is undergoing a gradual transition from conventional pollutant removal toward more sustainable management of water, materials, and energy [1,2]. Due to the increasing pressure on freshwater supplies, together with the growing complexity of wastewater streams, removal efficiency alone has proven insufficient as the sole criterion for evaluating treatment technologies [3]. Accordingly, sustainable chemical approaches are linking wastewater treatment with water reuse, resource recovery, and value creation. In this context, wastewater is regarded not simply as a waste stream, but as a potential source of water, chemicals, materials, and energy [4,5,6,7].

In our previous Special Issues, “Wastewater Treatment: Functional Materials and Advanced Technology” and the associated second edition, a broad range of functional materials and emerging technologies were collected, covering adsorption, ion exchange, membrane separation, advanced oxidation, electrochemical processes, and related approaches. These studies demonstrated substantial progress in the development of materials and processes for increasingly complex wastewater systems. At the same time, they also reflected an important evolution in this field: the focus of wastewater treatment is gradually expanding from maximizing pollutant removal toward reducing material and energy inputs, treating realistic water matrices, enabling water reuse, and extracting value from waste streams. The present Special Issue, “Sustainable Chemical Approaches for Wastewater Treatment and Resource Utilization,” was established against this background.

The five contributions collected in this Special Issue provide representative examples of this transition. One important research direction is developing sustainable treatment materials using renewable or waste-derived resources. Mariia Dmitrenko et al. developed mixed-matrix membranes based on carboxymethyl cellulose and carboxylated graphene oxide for pervaporation and nanofiltration [Contribution 1]. The combination of a bio-based polymer matrix with functional carbon materials enabled separation across different molecular scales, including applications involving dyes and heavy metal ions. This study demonstrates how renewable material platforms can be combined with functional modification to achieve effective and versatile water separation.

Biomass-derived carbon materials provide another direct link between waste valorization and environmental remediation. Tijana Marjanović Srebro et al. employed corn cob-derived biochar as a heterogeneous activator for persulfate oxidation of the organochlorine pesticides lindane and β-endosulfan [Contribution 2]. In addition to pollutant degradation, the study examined the underlying radical and non-radical pathways, catalyst reuse, and performance in real surface water. The transformation of agricultural residues into functional catalytic materials therefore provides a practical route for coupling biomass utilization with advanced water treatment.

In a complementary study, Nina Đukanović et al. systematically compared biochars derived from hardwood, corn cob, and wheat straw under different pyrolysis conditions [Contribution 3]. Their results revealed the important roles of biomass feedstock and thermal treatment in controlling pore structure, surface chemistry, and the resulting environmental applicability of biochar. Such structure–property relationships are important for moving biomass-derived materials from empirical utilization toward rational and application-oriented design.

A second direction represented in this collection is the integration of wastewater treatment with water reuse. Benan Yazıcı Karabulut investigated aluminum-based electrocoagulation for the treatment of real greywater and optimized the process using response surface methodology and kinetic modeling [Contribution 4]. Importantly, the treated water was considered in the context of non-potable reuse rather than pollutant removal alone. This work illustrates how wastewater treatment can contribute directly to water conservation and circular water management, particularly for decentralized or distributed treatment scenarios.

An even more explicit transition from pollutant removal to resource conversion is demonstrated by Patrick Niyirora et al. [Contribution 5]. In this work, rhenium-containing nanocomposite catalysts were incorporated into 3D-printed fixed-bed reactors for the continuous-flow reduction of nitroaromatic compounds to aromatic amines. Nitroaromatic compounds are important environmental contaminants, whereas their corresponding aromatic amines are valuable chemical intermediates. This concept is closely aligned with the emerging paradigm of pollutant valorization, in which organic contaminants in wastewater are no longer regarded solely as species to be degraded or mineralized, but as potential carbon resources that can be selectively converted into value-added chemicals, functional intermediates, or even polymeric materials through further coupling and polymerization processes [6,7]. From this perspective, wastewater treatment can evolve from contaminant destruction toward controlled molecular conversion and resource recovery. The use of a continuous-flow reactor in this study further strengthens the process-oriented relevance of such a treatment-to-valorization strategy.

Taken together, these contributions demonstrate that sustainable wastewater treatment requires consideration of the entire material and process cycle. Renewable and waste-derived feedstocks can be converted into treatment materials; wastewater can be reclaimed as an alternative water source; and selected contaminants can potentially be transformed into valuable chemical products. These developments expand the conventional boundaries of wastewater treatment and establish closer connections between environmental chemistry, materials science, separation science, catalysis, and chemical engineering.

Looking forward, the next stage of sustainable wastewater treatment should move beyond the optimization of individual removal processes toward the integration of pollutant control, resource recovery, and energy conversion.

Firstly, wastewater treatment can be coupled with energy-producing reactions. Electrochemical, photoelectrochemical, and related processes offer opportunities to couple anodic pollutant oxidation with cathodic hydrogen evolution, allowing contaminant removal and H_2_ production to occur within the same reaction system [8]. Likewise, microbial electrochemical and other bioelectrochemical processes may integrate wastewater treatment with electricity generation [9,10]. These strategies are conceptually different from simply reducing the energy consumption of wastewater treatment. Instead, they seek to transform treatment from a purely energy-consuming process into a platform capable of simultaneous environmental remediation and energy production.

Second, greater attention should be directed toward the selective recovery and valorization of wastewater constituents [11,12]. Organic contaminants can potentially serve as carbon feedstocks for the production of value-added chemicals, functional intermediates, or polymeric materials [6,7], while metals, phosphorus, nitrogen, and other inorganic components can be recovered as secondary resources [13,14,15]. Accordingly, complete destruction or mineralization may not always represent the most desirable treatment endpoint. Where technically and environmentally justified, selective transformation, separation, and recovery could provide greater value than conventional pollutant elimination.

Third, these emerging concepts must ultimately be translated from laboratory-scale demonstrations into continuously operated systems under realistic conditions. Long-term material stability, regeneration, energy and chemical consumption, secondary waste generation, product recovery and purity, and performance in complex real wastewater matrices all require systematic evaluation. Particular attention should also be paid to whether the proposed coupling of treatment, resource recovery, and energy or chemical production can remain effective under continuous operation. Life-cycle assessment and techno-economic analysis will therefore be essential for determining whether such multifunctional treatment processes can deliver genuine environmental and economic benefits on a practical scale [16].

Overall, wastewater treatment is expected to evolve from standalone purification technologies toward integrated water resource–energy systems, in which pollutant removal is coupled with water reuse, chemical production, resource recovery, hydrogen generation, or electricity production. Such integration provides an important pathway for reducing the environmental footprint of wastewater treatment while simultaneously recovering value from water and waste streams.

We hope that this Special Issue will stimulate further development of sustainable materials, selective chemical transformations, and integrated treatment processes, and will encourage closer connections between wastewater purification, resource utilization, and energy conversion.
